# The relationship between chest tube position in the thoracic cavity and treatment failure in patients with pleural infection: a retrospective cohort study

**DOI:** 10.1186/s12890-022-02157-x

**Published:** 2022-09-20

**Authors:** Jumpei Taniguchi, Kei Nakashima, Hiroki Matsui, Tatsuya Nagai, Ayumu Otsuki, Hiroyuki Ito, Hiroshi Sugimura

**Affiliations:** 1 Department of Pulmonology, Kameda Medical Center, 929 Higashi-cho, Kamogawa, Chiba 296-8602 Japan; 2 Department of Clinical Epidemiology and Health Economics, School of Public Health, The University of Tokyo, Tokyo, Bunkyou-ku Japan; 3 Clinical Research Support Office, Kameda Medical Center, Kamogawa, Chiba Japan; 4 Department of Thoracic Surgery, Kameda Medical Center, Kamogawa, Chiba Japan

**Keywords:** Chest tubes, Drainage, Empyema, Retrospective study, Pleural infection, Propensity score

## Abstract

**Background:**

Pleural infection is an infection of the pleural space that is usually treated with antibiotics and source control. Chest tube insertion is the most popular and widely used drainage technique. We typically attempt to place the tube at the bottom of the thoracic cavity to consider the effects of gravity; however, the effectiveness of this practice is not well-defined. Therefore, we aimed to examine whether the position of the tip of the thoracic tube affects treatment failure in patients with pleural infection.

**Methods:**

In this retrospective observational study, patients with pleural infection who underwent thoracic tube insertion were divided into two groups: those with the tip of the tube positioned below the 10th thoracic vertebra at the level of the diaphragm (lower position group) and those with the tip placed above the 9th thoracic vertebra (upper position group). We compared whether the position of the tube tip affected treatment failure. Stabilized inverse probability treatment weights (SIPTW) were used to balance the baseline characteristics between the groups. Treatment failure showed a composite outcome of hospital death, referral to surgeons for surgery, and additional chest tube insertion.

**Results:**

Among the 87 patients, 41 and 46 patients were in the lower and upper groups, respectively. No significant difference was observed in the composite outcomes between the groups (46.3% vs. 54.3%, *P* = 0.596). There was also no significant difference in the composite outcome between both groups after adjusting for SIPTW (52.3% vs. 68.8%, *P* = 0.286).

**Conclusions:**

There were no significant differences in the treatment failure in this study addressing pleural infection treatment, in which the drain tip position was stratified by the 9th and 10th thoracic vertebrae. The position of the tip of the thoracic tube may not be important for pleural infection treatment providing that it is in the thoracic cavity.

*Trial registration* The participants were registered retrospectively.

**Supplementary Information:**

The online version contains supplementary material available at 10.1186/s12890-022-02157-x.

## Background

Pleural infection is a common complication of pneumonia and often begins with pneumonia-associated pleural effusion [1]. Since the introduction of antibiotics, the overall incidence of pleural infection has declined dramatically [2]. However, epidemiologic studies suggest that the incidence rates are slowly rising [3]. Since pleural infection is associated with a 10–20% mortality rate, long hospital stays, and a heavy financial burden [4–6], it remains an important clinical challenge.

The two mainstays of pleural infection treatment are prompt antibiotic initiation and appropriate source control [1]. Parapneumonic effusion and empyema related to pleural infection are divided into three stages: Stage I (acute exudative stage), Stage II (subacute fibrinopurulent stage), and Stage III (chronic organizing stage) [7]. For patients with small uncomplicated parapneumonic effusions (stage I), drainage might not be necessary unless the effusion is sizeable enough to impair the respiratory function. However, for patients with complicated parapneumonic effusions (stage II), drainage should be immediately performed for source control, and in a later stage (stage III), surgery might be required [1]. To date, in addition to administering antibiotics, chest tube insertion is the least invasive, most widespread drainage method, and is considered the gold standard treatment approach for pleural infection [8]. Inadequate improvement after the intake of antibiotic and thoracic drainage tube placement indicates inadequate drainage, and various nonsurgical options can be considered, such as additional drainage procedures and/or intrapleural fibrinolytic agents [1]. Surgical intervention is the last resource that attempts to drain pus and expand the lung with video-assisted thoracic surgery or open thoracotomy [9]. Treatment failure after chest drainage tube placement is associated with long hospital stays, financial burden, and high mortality [10, 11]; thus, it is important to reduce the failure rate of treatment with tube placement.

Proper placement of the tube in the thoracic cavity is an important factor for successful treatment, and insertion is usually performed using ultrasound, X-rays, and computed tomography (CT) for image guidance [12]. Immediately following drainage, patients typically undergo a chest X-ray for rudimentary assessment of the tube or catheter placement. Although it is important that the tip of the drainage tube containing the drainage holes that tap purulent fluid is placed in the thoracic cavity [13], there is no consensus on the position of the drain tip. Previous guidelines have recommended that drain tips for fluid accumulation should ideally be placed at the base of the thoracic cavity [14]. This method might reflect the fact that fluid tends to accumulate in the basal region of the thoracic cavity owing to the effect of gravity, and this practice is still customary. To date, there is no clear evidence that this practice is useful in pyothorax, which can lead to not only free-flowing or unilocular effusions (i.e., effusion without internal septa), but also complex effusions (i.e., effusions with internal septations or locules).

In this study, we retrospectively examined the position of the tip of the drainage tube in patients with pleural infection to determine whether the drain tip position resulted in a difference with respect to treatment failure of pleural infection.

## Methods

### Study population

To evaluate whether the drain tip position of the chest tube affects treatment failure, we focused in this study on patients with stage II or early-stage III acute pleural infection where drainage therapy would be most effective. We retrospectively enrolled consecutive patients (≥ 18 years) who underwent chest tube placement for pleural infection between January 2011 and July 2021 at the 917-bed Kameda Medical Center in Japan. The inclusion criteria were patients: (1) who were hospitalized (≥ 18 years old); (2) with an International Classification of Diseases 10th revision (ICD-10) diagnosis of pyothorax without fistula (J869) on admission (cases in which the clinician diagnosed stage II–III pleural infection based on the characteristics of the pleural effusion and imaging study); and (3) who underwent continuous thoracic drainage or percutaneous empyema drainage during hospitalization (coded as J019, K496-5 in the Japanese original codes). The exclusion criteria were patients having: (1) postoperative pleural infection (developed pleural infection within 1 month after surgery); (2) traumatic pleural infection; (3) pleural infection with malignant pleural effusion; (4) chronic empyema (patients with highly organized pleural effusion and/or fibrinous pleural covering); (5) recurrence within 3 months of treatment; (6) multiple tube insertion at the initial treatment; and (7) small, localized pleural effusion that does not extend across the 9th and 10th thoracic vertebrae. The following patient demographics and clinical variables were collected: age, sex, body mass index (BMI), blood laboratory data on admission, pleural fluid analysis at thoracentesis or chest tube placement, and imaging data. This retrospective cohort study was reviewed and approved by the Research Ethics Committee of Kameda Medical Center (#21-091). The requirement for written informed consent was waived due to the retrospective nature of the study.

### Management

All patients were initiated on empiric broad-spectrum intravenous antibiotic therapy once thoracic pleural infection was identified. Once the causal bacteria were cultured and identified, the antibiotics were modified based on the antimicrobial susceptibility test findings. The type of medical professionals performing the pleural drainage; the techniques, drain types, and sizes used; and the sonography-guided thoracentesis before chest tube insertion were decided by the attending physicians, based on their assessment of the patient's needs. In our hospital, thin tubes (≤ 14 Fr) tended to be selected for X-ray/CT guidance. In contrast, medium-sized tubes (16–24 Fr) were used in the absence of X-ray/CT guidance. The chest tube drainage system used traditional three-chamber plastic units [15]. After the chest tube was inserted, 0 to − 20 cm of water suction level was used depending on the drainage volume, and some patients were treated with urokinase at the discretion of their physicians. Only urokinase was available at our hospital, thus other intrapleural fibrinolytic agents, such as streptokinase and tissue plasminogen activator (t-PA), were not used. Failure to improve after the administration of antibiotics and tube thoracostomy drainage (e.g., persistent or worsened effusion, persistent or new fever, persistent or worsening leukocytosis, or persistently elevated inflammatory markers) resulted in patients undergoing additional drainage procedures and/or were referred to a surgeon for surgery.

### Position of the tip of thoracic tube

**Fig. 1:**
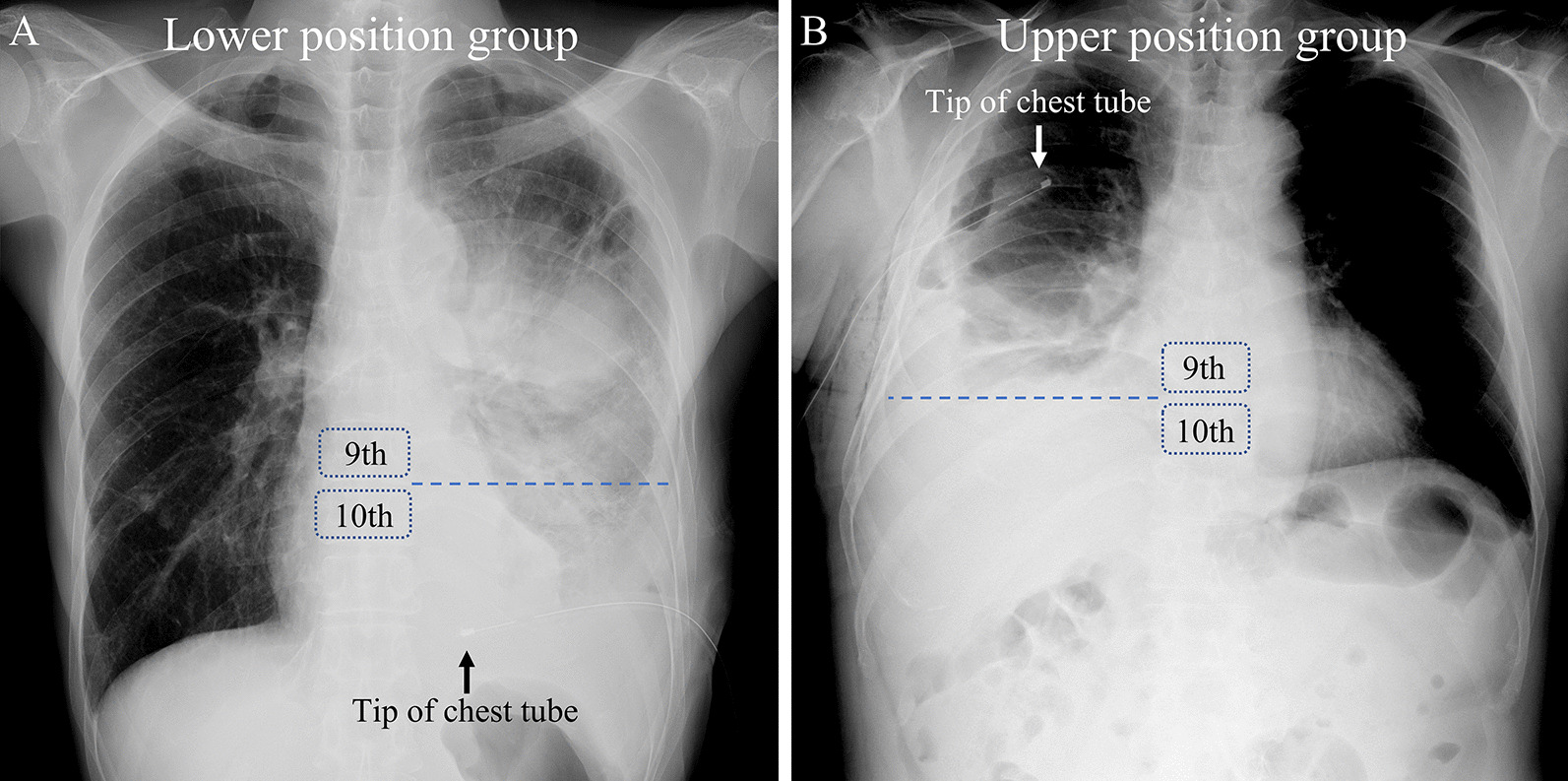
Chest X-ray highlighting the 9th and 10th thoracic vertebrae and the tip of chest tube. **A** Assigned to the lower position group. **B** Assigned to the upper position group

The exposure in this study was the position of the tip of the drain tube. The drain tip position in the thoracic cavity was determined using chest X-ray performed after insertion. Good inspiration on a chest radiograph showed at least 9 posterior ribs [16]. In this study, patients who had their drain tip positioned below the 10th thoracic vertebra, which is assumed to correspond to the level of the diaphragm, comprised the lower position group, and those who had their drain tip positioned above the 9th thoracic vertebra comprised the upper position group. A chest radiograph highlighting the 9th and 10th thoracic vertebra and chest tube is shown in Fig. 1.

### RAPID score

The RAPID score is a clinical risk prediction score in adults with pleural infection and corresponds to renal (urea), age, fluid purulence, infection source (hospital vs. community), and dietary factors (albumin) [RAPID] [17]. The RAPID score at baseline was calculated according to the parameters shown in Additional file 1: Supplementary Table 1, as defined in the original article [17]. Patients were assigned to one of three risk categories according to their score (low-risk [score 0–2], medium-risk [score 3–4], and high-risk [score 5–7]). Recently, it has been suggested that this RAPID score category is useful for predicting mortality in several studies [18, 19].

### Outcomes

Treatment failure was defined as a composite outcome of hospital death, referral to surgeons for surgery, and additional chest tube insertion.

### Statistical analyses

We obtained the demographic statistics of patients and compared them with exposure. Statistical analyses were performed using the Wilcoxon rank-sum test for continuous variables and Chi-square test for categorical variables. Clinical outcomes were also compared according to exposure using the Chi-square test.

We adjusted the backgrounds of the patients using stabilized inverse probability treatment weights and estimated the treatment effect of exposure. We calculated the stabilized inverse probability of treatment weights (SIPTW) using logistic regression for exposure using confounders (age, sex, BMI, C-reactive protein [CRP], pleural-fluid characteristics [culture positive for bacteria, pH, glucose], lactate dehydrogenase [LDH], X-ray/CT-guided chest tube insertion, and RAPID score) as predicting variables [12, 17, 20, 21]. After weighting, we measured the differences between each group using standardized mean differences (SMD) for the covariates. An SMD lower than 0.1 indicated a good balancing of the covariate [22]. Statistical analyses were performed using R software (version 3.6.3, R Development Core Team, https://www.r-project.org/).

## Results

**Fig. 2:**
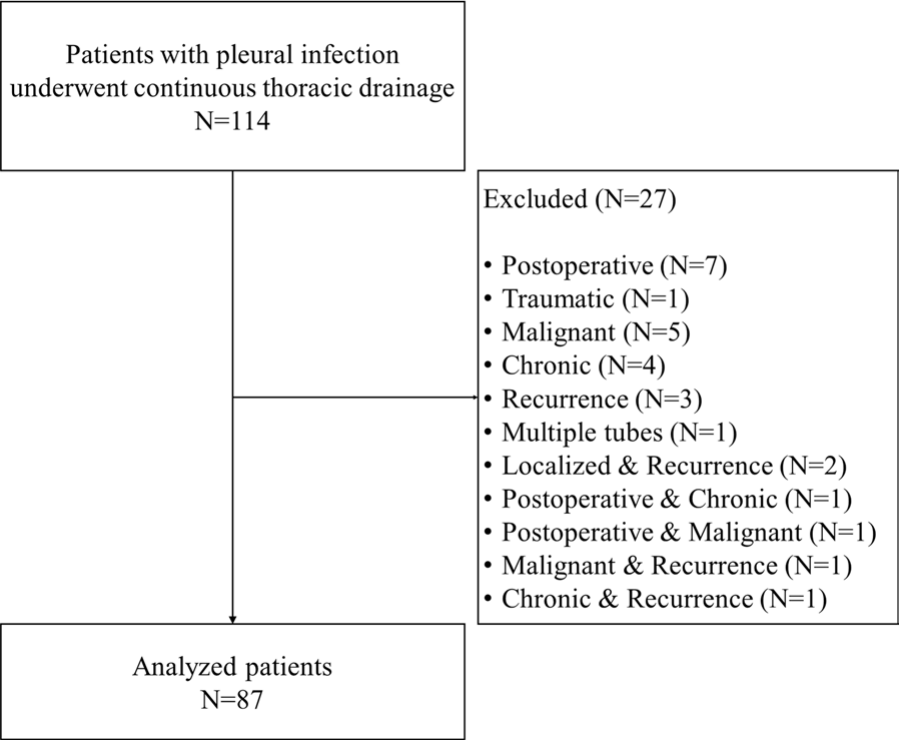
Flowchart for selection of patients

Figure 2 shows the patient selection flowchart. A total of 114 patients were included in this study, and 27 patients were excluded for the reasons shown in Fig. 2. Therefore, the final study sample comprised 87 patients.

The demographic and clinical unweighted and weighted characteristics of the eligible patients, stratified according to the position of the tip of the thoracic tube, are summarized in Table 1. In the unweighted patient characteristics, the lower position group had a significantly higher median age of 71.0 years old (66.0–82.0), compared to the upper position group, which had a median age of 68.0 years old (58.8–74.0). However, there were no significant differences in sex, BMI, CRP level, and positive culture of pleural fluid between the two groups. The median pleural fluid pH, glucose, and LDH levels were 7.33 (interquartile range [IQR]: 7.21–7.50), 19.0 mg/dL (IQR: 1.0–83.0), and 1267.5 U/L (IQR: 495.0–2077.5) in the lower position group, and 7.32 (IQR: 7.13–7.49), 27.0 mg/dL (IQR: 1.0–71.0), and 1697.0 U/L (IQR: 973.0–3595.0) in the upper position group, respectively, and there was no significant difference between the two groups. The use of urokinase, X-ray/CT-guided chest tube insertion, and the size of the chest tube were determined at the discretion of the physician, but there was no significant difference between the two groups. The RAPID scores were also not significantly different between the two groups. Following SIPTW adjustment, the SMD of age, sex, CRP, X-ray/CT-guided chest tube insertion, and pleural fluid characteristics (culture positive for bacteria, pH, glucose, and LDH) were < 10%. However, SMD in BMI, urokinase level, chest tube size, and RAPID score exceeded 10%; these confounders were imbalanced.

**Table 1:** Patient characteristics classified by position of the tip of thoracic tube in unweighted and weighted study populations

	Unweighted study population				Weighted study population			
	Lower position group (n = 41)	Upper position group (n = 46)	*p* value	SMD	Lower position group (n = 35.3)	Upper position group (n = 27.6)	*p* value	SMD
Age, years (median ± IQR)	71.0 (66.0–82.0)	68.0 (58.8–74.0)	0.029	0.490	70.0 (61.2–79.6)	72.0 (64.4–76.3)	0.839	0.044
Females, n (%)	2 (4.9)	8 (17.4)	0.136	0.406	6.6 (18.8)	4.4 (15.8)	0.835	0.079
BMI, kg/m^2^ (median ± IQR)	19.7 (17.2–23.9)	21.3 (18.3–25.2)	0.121	0.321	19.6 (16.3–23.9)	20.3 (17.9–23.6)	0.756	0.222
CRP, mg/dL (median ± IQR)	18.3 (10.7–22.9)	19.6 (12.8–30.1)	0.308	0.257	19.5 (13.1–26.4)	16.8 (11.6–24.5)	0.365	0.030
Urokinase use, n (%)	30 (73.2)	34 (73.9)	1.000	0.017	26.0 (73.5)	21.5 (77.8)	0.756	0.101
Pleural-fluid characteristic								
Culture positive for bacteria, n (%)	19 (51.4)	25 (58.1)	0.702	0.137	21.1 (59.8)	15.8 (57.2)	0.863	0.052
pH, (median ± IQR)	7.33 (7.21–7.50)	7.32 (7.13–7.49)	0.659	0.108	7.27 (7.08–7.52)	7.26 (7.11–7.49)	0.963	0.036
Glucose, mg/dL (median ± IQR)	19.0 (1.0–83.0)	27.0 (1.0–71.0)	0.741	0.002	10.2 (1.0–86.6)	3.0 (1.0–80.2)	0.778	0.002
LDH, IU/mL (median ± IQR)	1267.5 (495.0–2077.5)	1697.0 (973.0–3595.0)	0.065	0.306	1093.2 (540.9–1727.4)	1407.2 (919.3–2966.4)	0.300	0.061
X-ray/CT-guided chest tube insertion, n (%)	7 (17.1)	10 (21.7)	0.782	0.118	6.3 (17.8)	4.9 (17.8)	0.997	0.001
Chest tube size, French			0.894	0.104			0.794	0.192
≤ 14, n (%)	9 (22.5)	9 (20.5)			7.2 (20.4)	4.0 (14.3)		
15–20, n (%)	21 (52.5)	22 (50.0)			15.2 (43.1)	14.1 (51.2)		
> 20, n (%)	10 (25.0)	13 (29.5)			12.9 (36.5)	9.5 (34.4)		
RAPID score			0.232	0.374			0.841	0.181
Low risk, n (%)	8 (19.5)	16 (34.8)			13.2 (37.3)	8.0 (28.9)		
Medium risk, n (%)	23 (56.1)	23 (50.0)			14.6 (41.3)	13.2 (47.9)		
High risk, n (%)	10 (24.4)	7 (15.2)			7.6 (21.4)	6.4 (23.2)		

*IQR* interquartile range

Outcomes stratified according to the position of the thoracic tube tip are shown in Table 2. The unweighted composite outcome was 19 (46.3%) in the lower position group and 25 (54.3%) in the upper position group, with no significant difference between the two groups. There were 4 deaths in the lower position group and 2 deaths in the upper position group. Surgical procedures and additional chest tube insertions were performed in 8 and 10 patients in the lower position group, and in 12 and 13 patients in the upper position group, respectively. The breakdown of each outcome (death during hospitalization, referral surgeons for surgery, and additional chest tube insertion) also did not significantly differ between the two groups.

**Table 2:** Clinical outcomes classified by position of the tip of thoracic tube in the unweighted and weighted study populations

	Unweighted study population				Weighted study population			
	Lower position group (n = 41)	Upper position group (n = 46)	*p* value	SMD	Lower position group (n = 35.3)	Upper position group (n = 27.6)	*p* value	SMD
Composite outcome^†^, n (%)	19 (46.3)	25 (54.3)	0.596	0.161	18.5 (52.3)	19.0 (68.8)	0.286	0.343
Death during hospitalization, n (%)	4 (9.8)	2 (4.3)	0.569	0.212	3.0 (8.4)	1.0 (3.6)	0.435	0.205
Surgery, n (%)	8 (19.5)	12 (26.1)	0.637	0.157	11.9 (33.8)	7.4 (26.7)	0.648	0.155
Additional chest tube insertion, n (%)	10 (24.4)	13 (28.3)	0.869	0.088	8.0 (22.6)	12.5 (45.3)	0.128	0.494

^†^Composite outcome: death, surgery, or an additional chest tube insertion

There were no significant differences in any outcome, including the composite outcome, in the weighted study population.

## Discussion

In this study, we evaluated the effect of chest tube position on treatment failure. Our study findings, which were derived from 87 patients with pleural infection, suggest that chest tube drain tip positioning below the 10th or above the 9th thoracic vertebra, had no effect on treatment failure. To the best of our knowledge, this is the first study to evaluate the relationship between the level of the chest tube tip positioning and pleural infection treatment failure.

Our findings have important clinical implications for physicians treating pleural infection. Past guidelines and the idea that fluid collects in the most dependent portion suggest that chest tube drains are most effective when placed in the lowest portion of the thoracic cavity [14]. Clinicians typically insert a chest tube through the lower intercostal space, and once verified to be in the thoracic cavity, it is pushed down to the level of the diaphragm so that it is optimally placed in the inferior and most dependent portion of the chest.

Tube malposition is the most common complication of tube thoracostomy [23]. Drain insertion under image guidance has been proven to be effective and is therefore now being widely practiced [24]. However, as shown in our study, if the only important factor is the proper insertion of the drain tube without malposition, where the height of the tip does not affect treatment failure for pleural infection, then it can lead to reduction in procedure time on targeting the bottom in the thoracic cavity and avoidance of complications (organ injury, e.g., liver, spleen) caused by aiming at a lower position in the thoracic cavity.

There are several possible reasons why the level of the drain tip did not affect treatment failure in this study. First, the position of fluid retention depends on the patient's position [25]. Usually, hospitalized patients with chest tube drains are in bed rest because of pain. Because pleural effusions accumulate in a gravity-dependent manner, they may move dorsally or laterally rather than at a lower position, depending on the position of the patient. This position-dependent migration of pleural fluid within the thoracic cavity might be the reason why the level of the drain tip did not affect treatment failure. The second reason is the high rate of urokinase usage. Urokinase is a fibrinolytic enzyme which breaks down fibrinous adhesions that are part of the organization process and are responsible for the encapsulated pus [26]. In this study, urokinase was used in more than 70% of patients in both groups, which may have decreased the viscosity of the pleural effusion and facilitated drainage independent of the height of the drainage tube.

This study has several limitations. First, this was a retrospective single-center study. Because this study did not follow a standardized protocol, the selection of antibiotics, techniques, drain types and sizes used, type of the medical personnel performing the pleural drainage, urokinase use, and selection of next treatment options in case of treatment failure were determined according to the judgement of the individual needs of each patient by the treating physician. These factors may preclude the extrapolation of our conclusions to other facilities. Second, in this research, we excluded patients with small, localized pleural effusion that does not extend across the 9th and 10th thoracic vertebrae. Therefore, the study results cannot be applied to cases where pleural effusion is heavily loculated in the upper to the middle part of the thoracic cavity in which placement of the tip of the tube just above the diaphragm is impossible. Third, in this study, we defined the lower position group as those with the tip of the drain tube positioned below the 10th thoracic vertebra, which is generally considered to be just above the diaphragm. This is the first study to examine the influence of the level of the drain tip in the thoracic cavity on treatment failure in pleural infection, thus the validity of this definition has not yet been clearly established. Ideally, the drain tip position should be measured at each thoracic vertebral height in many patients to confirm that the drain tip position does not contribute to treatment failure. Fourth, even after weighting adjustment, some confounding factors could not be balanced. The lower-position group had a low BMI and a high RAPID score. As people with a low BMI and high RAPID score tend to be skinnier and frailer, the lower intercostal spaces are more easily identifiable. A low BMI and high RAPID score were associated with poor prognosis, which may have influenced our results [19, 27]. If the study was conducted with a larger sample size and the patients were well balanced in both groups, placement of the drain tip in a lower position might have been correlated with lower treatment failure. Therefore, a prospective multicenter randomized controlled trial with a standardized protocol and larger sample size is needed to confirm our results.

## Conclusions

In this study, there were no significant differences in treatment failure of pleural infection when the chest tube drain tip was positioned below the 10th or above the 9th thoracic vertebrae. Our findings suggest that chest tube drain tip position may not be crucial to treatment success as assumed. Nonetheless, further multicenter prospective studies with standardized protocols are required to confirm our results.

## Supplementary Information

**Additional file 1.** **Supplementary Table 1.** The RAPID scoring system using baseline parameters.

## Data Availability

The data supporting the findings of this study are available from the corresponding author upon reasonable request. The data are not publicly available because of privacy and ethical restrictions.

## Abbreviations

SIPTW: Stabilized inverse probability treatment weights
IQR: Interquartile range
BMI: Body mass index
SMD: Standardized mean difference
CRP: C-reactive protein
LDH: Lactate dehydrogenase
RAPID: Renal, age, fluid purulence, infection source, and dietary factors
