# Supplementary material for: The relationship between chest tube position in the thoracic cavity and treatment failure in patients with pleural infection: a retrospective cohort study
Source: BMC Pulm Med. 2022 Sep 20;22:358. doi: 10.1186/s12890-022-02157-x (PMC9490893; doi:10.1186/s12890-022-02157-x)
Supplement: Supplementary file 1 — Additional file 1. Supplementary Table 1. The RAPID scoring system using baseline parameters. [file 12890_2022_2157_MOESM1_ESM.docx]

Supplementary Table 1. The RAPID scoring system using baseline parameters

| Parameter | Measure | Score |
| --- | --- | --- |
| Renal, urea (mmol/L) | < 5.0 | 0 |
|  | 5.0–8.0 | 1 |
|  | > 8.0 | 2 |
| Age (years) | < 50 | 0 |
|  | 50–70 | 1 |
|  | > 70 | 2 |
| Purulence of pleural fluid | Purulent | 0 |
|  | Non-purulent | 1 |
| Infection source | Community-acquired | 0 |
|  | Hospital-acquired | 1 |
| Dietary factor, albumin (g/L) | ≥ 27.0 | 0 |
|  | < 27.0 | 1 |

Source: Rahman NM, Kahan BC, Miller RF, Gleeson FV, Nunn AJ, Maskell NA. A Clinical Score (RAPID) to Identify Those at Risk for Poor Outcome at Presentation in Patients with Pleural Infection. Chest. 2014;145(4):848-55.
